# Supplementary figures and images for: Intersubject MVPD: Empirical comparison of fMRI denoising methods for connectivity analysis
Source: PLoS One. 2019 Sep 24;14(9):e0222914. doi: 10.1371/journal.pone.0222914 (PMC6759145; doi:10.1371/journal.pone.0222914)

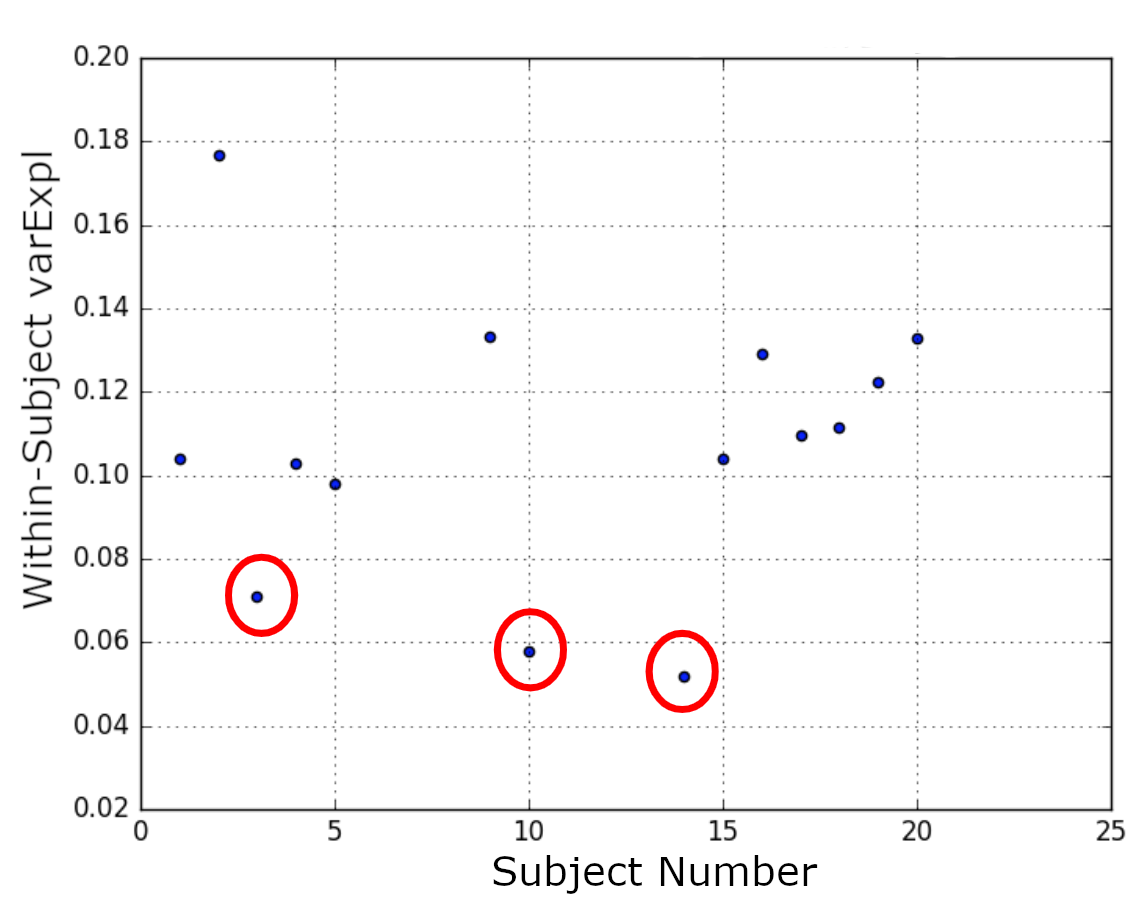

Supplement: S1 Fig — The figure shows the within-subject prediction accuracy of all subjects who completed all the experimental sessions and passed the FMRIPREP preprocessing pipeline. Each dot shows the mean variance explained across all region pairs within a subject using a default denoising approach consisting of removal of scanner drift followed by CompCorr. Three outlier subjects were identified due to their low within-subject prediction accuracy. (TIF) [file pone.0222914.s001.tif]

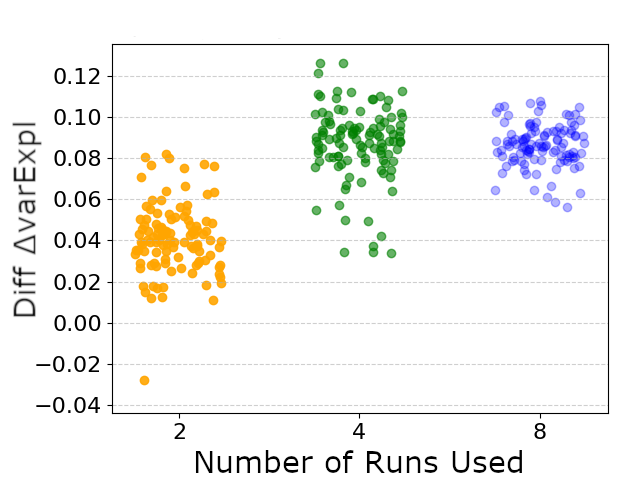

Supplement: S2 Fig — The figure shows the differences between the discrepancy metric obtained with no denoising, and the discrepancy metric obtained with removal of global signal and CompCorr using different amount of data. Each dot represents a subject pair. From left to right: yellow dots show the differences using one quarter of the data (2 runs), green dots show the differences using half of the data (4 runs), and blue dots show the differences using full data (8 runs). (TIF) [file pone.0222914.s002.tif]
